# Transfer efficiency of carbon, nutrients, and polyunsaturated fatty acids in planktonic food webs under different environmental conditions

**DOI:** 10.1002/ece3.7651

**Published:** 2021-05-18

**Authors:** Maciej Karpowicz, Irina Feniova, Michail I. Gladyshev, Jolanta Ejsmont‐Karabin, Andrzej Górniak, Nadezhda N. Sushchik, Olesya V. Anishchenko, Andrew R. Dzialowski

**Affiliations:** ^1^ Department of Hydrobiology Faculty of Biology University of Białystok Białystok Poland; ^2^ Institute of Ecology and Evolution Russian Academy of Sciences Moscow Russia; ^3^ Institute of Biophysics of Federal Research Centre Krasnoyarsk Science Centre of Siberian Branch of Russian Academy of Sciences Krasnoyarsk Russia; ^4^ Siberian Federal University Krasnoyarsk Russia; ^5^ Research Station in Mikołajki Nencki Institute of Experimental Biology Polish Academy of Sciences Warsaw Poland; ^6^ Department of Integrative Biology Oklahoma State University Stillwater OK USA

**Keywords:** biogeochemical cycle, dystrophication, essential substances, eutrophication, food quality, phytoplankton, zooplankton

## Abstract

The trophic transfer efficiency (TTE) is an important indicator of ecosystem functioning. However, TTE data from freshwater food webs are ambiguous due to differences in time scales and methods. We investigated the transfer of essential substances (carbon, nutrients, and polyunsaturated fatty acids) through plankton communities in 30 Polish lakes with different trophic status in the middle of summer. The results of our study revealed that different essential substances were transferred from phytoplankton to zooplankton with varying efficiencies. The average TTE of C, N, P, and the sum of ω‐3 PUFA were 6.55%, 9.82%, 15.82%, and 20.90%, respectively. Our results also show a large mismatch between the elemental and biochemical compositions of zooplankton and their food during the peak of the summer stagnation, which may further promote the accumulation of essential substances. There were also large differences in TTEs between trophic conditions, with the highest efficiencies in oligotrophic lakes and the lowest in dystrophic and eutrophic lakes. Therefore, our study indicates that disturbances like eutrophication and dystrophication similarly decrease the TTE of essential substances between phytoplankton and zooplankton in freshwater food webs.

## Graphical abstract

The results of our study revealed that different essential substances (C, N, P, and polyunsaturated fatty acids) were transferred from phytoplankton to zooplankton with varying efficiencies. There were also large differences in TTEs between trophic conditions, with the highest efficiencies in oligotrophic lakes and the lowest in dystrophic and eutrophic lakes. Therefore, our study indicates that disturbances like eutrophication and dystrophication similarly decrease the TTE of essential substances between phytoplankton and zooplankton in freshwater food webs.

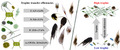

## 1 INTRODUCTION

Interactions at the phytoplankton–zooplankton interface are important for the overall functioning of lake ecosystems. There is often a mismatch in the elemental and biochemical composition between primary producers and consumers (Feniova et al., 2018; Hessen, 1992; White, 1993). Food quality commonly does not meet the requirements of herbivorous consumers in freshwater environments because of nutrient deficiency (Plath & Boersma, 2001). The food resources for zooplankton consist of a heterogeneous mixture of phytoplankton, bacteria, and other particles collectively labeled “seston,” and as a result, the carbon:phosphorus (C:P) ratio in seston can vary greatly from 100 to 1,000 (Hessen et al., 2013). Furthermore, different algal taxa have different C:N:P ratios. For example, Chlorophyceae seem to be evolutionary geared toward higher C:P compared with other groups (Quigg et al., 2003). Different phytoplankton species also vary in polyunsaturated fatty acid (PUFA) composition and their elemental ratios of C:P, C:N, N:P (Gladyshev et al., 2007). In contrast, C:P ratios of zooplankton are nearly constant at 80:1 (Elser et al., 2000). This mismatch in elemental ratios between producers (high C:P and C:N) and consumers (low C:P and C:N) may have a negative impact on the fitness of individual consumers and can alter the synthesis of macromolecules such as lipids, proteins, and nucleic acids (Anderson et al., 2004; Elser et al., 2000; Prater et al., 2018; Sterner & Elser, 2002).

According to the threshold elemental ratio model, it is assumed that the element in the least supply is assimilated with maximum efficiency. In the case of *Daphnia*, phosphorus assimilation is often set to 1 (100%), while carbon is 0.6 and decreases to almost zero with increasing seston C:P ratio (Hessen et al., 2013; Olsen et al., 1986). Laboratory experiments have shown that regeneration of nutrients can be calculated with high accuracy at low and high food quality, but most of them refer to *Daphnia* species (Attayde & Hansson, 1999; Hall, 2009). Sterner et al. (1992) present a striking example of linking the classical trophic cascade to stoichiometric‐driven effects in food webs, where a shift from large cladocerans (low N: P) to copepods (high N: P) caused a shift from P to N limitation for autotrophs. Thus, seston quality is very important for zooplankton, their community composition, nutrient cycling, and efficiency of transfer of energy and matter (Hessen et al., 2013). In summer, the proportion of inedible phytoplankton increases while food quality decreases (Sommer et al., 2012), thus negatively affecting zooplankton development. Therefore, it is especially important to understand how such mismatches between primary producers and consumers affects the transfer of essential substances in planktonic food webs. For this reason, we investigated the transfer of carbon, nutrients, and PUFAs through plankton communities in 30 lakes with different trophic status in the middle of the summer stagnation.

Production and efficiency of matter transfer are usually measured in carbon units because phytoplankton converts carbon from inorganic to organic, which is incorporated into the biomass of zooplankton (Pauly & Christensen, 1995; Schulz et al., 2004). A major paradigm of ecology is that only about 10% of organic carbon production of one trophic level is incorporated into new biomass in the next trophic level (Lindeman, 1942). Nevertheless, there are large discrepancies in trophic transfer efficiency (TTE) from phytoplankton to zooplankton in freshwater ecosystems. Data based on annual primary and secondary production suggest that TTE varies around 5%–10% (Gladyshev et al., 2011; Lacroix et al., 1999; Schulz et al., 2004). However, weekly analysis in a small Siberian reservoir indicated that TTE during the peak of the summer stagnation can be much lower (Gladyshev et al., 2011). Radio‐tracer experiments showed that TTE in a "microbial loop" is even lower, where only about 1–2 percent of bacterial production was present in larger organisms (Ducklow et al., 1986; Koshikawa et al., 1996). Thus, the TTE of carbon is highly variable in freshwater lakes, and there is evidence that efficient systems can support 25 times more zooplankton biomass than highly eutrophic lakes (Brett & Müller‐Navarra, 1997). There is also evidence that nutrients can effectively accumulate in zooplankton when food quality is low (Hessen et al., 2013; Olsen et al., 1986). For instance, polyunsaturated fatty acids of ω‐3 family with 18–22 carbon atoms (PUFA), which are essential for zooplankton (Arts et al., 2001; Wacker & Von Elert, 2001), were transferred from producers to primary consumers with about two times higher efficiency than bulk carbon (Gladyshev et al., 2011). Under low primary production, most of the ω‐3 PUFAs should be accumulated by the zooplankton, while nonessential C16‐PUFA are preferentially oxidized by the zooplankton (Brett et al., 2006; Gladyshev et al., 2011). Zooplankton can use ω‐3 PUFAs for catabolism, but only when there is excess PUFA under high primary production (Gladyshev et al., 2011). Thus, the transfer of essential substances in planktonic food webs can be highly variable and depend on the elemental and biochemical composition, community structure, and trophic status.

The aim of this study was to compare the transfer efficiencies of essential substances (C, N, P, and ω‐3 PUFA) from phytoplankton to zooplankton. We performed a snapshot survey in 30 lakes in the middle of summer to evaluate the effect of different trophic conditions and community composition on the TTE in planktonic food webs. We also studied how mismatches in biochemical composition between primary producers and primary consumers affected the biochemical cycles of essential elements. The coupling of nutrient and carbon cycles via their biotic interactions is especially important when considering global warming and human activity that intensify eutrophication in freshwater lakes. We expect that different essential substances would be transferred from phytoplankton to zooplankton with different efficiencies due to different trophic conditions and community composition. We also expect that eutrophication and dystrophication processes decrease the TTE of essential substances in planktonic food webs.

## 2 METHODS

### 2.1 Study sites and trophic characteristics

The study was carried out in 30 lakes in NE Poland with various trophic conditions and morphometric parameters (Tables 1 and S1). The trophic status was evaluated based on Carlson trophic state index (TSI) as an average of three equations, which include Secchi disk visibility (SDV), chlorophyll *a*, and total phosphorus (Carlson, 1977). Dystrophic conditions were detected by the hydrochemical dystrophy index (HDI), which uses data on surface water pH, electric conductivity, and DIC/DOC ratio (Górniak, 2017). The TSI of the studied lakes ranged from 29.6 to 63.8, and there were 5 oligotrophic lakes (no. 1–5), 7 mesotrophic lakes (no. 6–12), 8 eutrophic lakes (no. 13–20), and 10 dystrophic (humic) lakes (no. 21–30) (Table S1).

**TABLE 1:** The average ±standard deviation of morphometric and trophic parameters of the 30 study lakes based on their trophic status

	Oligotrophic	Mesotrophic	Eutrophic	Dystrophic
Surface (ha)	374.4 ± 252.4	291.4 ± 182.9	536.3 ± 605.0	5.4 ± 3.6
Max depth (m)	46.8 ± 7.8	29.3 ± 10.9	31.3 ± 12.7	5.0 ± 2.0
Average depth (m)	14.2 ± 2.2	9.5 ± 3.2	8.0 ± 3.2	–
Secchi disk visibility (m)	5.6 ± 1.5	2.5 ± 1.1	1.3 ± 0.6	1.3 ± 0.5
Chlorophyll *a* (µg/L)	9.2 ± 13.2	7.5 ± 2.3	29.2 ± 21.9	70.2 ± 37.8
DO in hypolimnion (mg/L)	6.2 ± 2.4	2.8 ± 2.4	0.2 ± 0.4	0.2 ± 0.2

The oligotrophic, mesotrophic, and eutrophic lakes were large and deep, while the dystrophic lakes where small and shallow. The average morphometric and trophic parameters are presented in Tables 1 and S1. The dystrophic lakes were distinguished by high HDI values from 60.4 to 79.9 (Table S1), which indicated advanced dystrophy. These lakes are usually oval and isolated with a catchment area covered with forests. The other distinguishing features of dystrophic lakes are acidic water, small concentrations of dissolved mineral substances, but with large concentrations of DOC and humic substances (Górniak, 2017; Karpowicz, Ejsmont‐Karabin, et al., 2020). Dystrophic lakes also have a yellow‐brown water color that results in a unique light climate and rapid warming of the epilimnion leading to strong thermal and oxygen stratification. Anoxic conditions in these lakes can be observed even at depth of 1 m (Karpowicz, Ejsmont‐Karabin, et al., 2020).

### 2.2 Field study and sampling

The field study and sampling were performed in the middle of summer 2019 (from July 22 to August 1). The sampling stations were located close to the deepest point in each lake (Table S1). The field measurements included SDV, temperature, concentration of oxygen, electrical conductivity, and pH using an HQ40D Multi Meter (Hach‐Lange GmbH). The submersible spectrofluorometer FluoroProbe (bbe‐Moldaenke) was used to obtain in situ measurements of total chlorophyll *a* and four phytoplankton classes: green algae, cyanobacteria, diatoms, and cryptophytes. Photosynthetic active radiation (PAR) was measured with the LI‐193SA Spectral Quantum sensor (LI‐COR Biosciences).

Water samples were collected using a 5‐L Limnos sampler from the different layers (epilimnion, metalimnion, and hypolimnion) of each lake. Quantitative samples of zooplankton for microscopic analysis were taken from each layer, where ten liters of water was filtered through a 50 µm plankton net and fixed with 4% formalin. We also collected samples of seston and zooplankton for elemental and fatty acids analyses. The seston contains inorganic particles and live organisms (phytoplankton, small rotifers, bacteria, etc.) that passed through a net with 100 µm mesh, but generally, the main component of seston in freshwater lakes is phytoplankton (Feniova et al., 2018). For the seston samples, we collected 0.5–2.0 L of water and filtered it through precombusted glass‐fiber GF/F filters (Whatman). Zooplankton for elemental and fatty acids analyses were collected by vertical net hauls and filtered on mesh sieves (100 µm). Then, zooplankton were dried on filter paper. Each zooplankton sample was divided into subsamples for fatty acids and elemental analyses. The zooplankton samples were dried over 24 hr at 75°C, while GF/C filters for seston analyses were dried at room temperature for 24 hr. Seston and zooplankton samples were then stored in a desiccator until further analyses as described below.

### 2.3 Laboratory analyses

Rotifer and crustacean species were identified and counted in the entire volume of each sample. Additionally, the lengths of 10 individuals of each species were measured and used to estimate the wet weight of crustaceans by applying equations from Błędzki and Rybak (2016). The biomass of rotifers was calculated based on length‐weight relationships proposed by Ejsmont‐Karabin (1998).

The chemical analyses of the water were conducted in the laboratory immediately after collection. Total phosphorus (TP) analyses were conducted according to the conventional photocolorimetric method (Murphy & Riley, 1962), modified by Neal et al. (2000). The concentrations of dissolved organic carbon (DOC), dissolved inorganic carbon (DIC), and total carbon (TC) were analyzed in the TOC‐L Series analyzers (Shimadzu).

The carbon and nitrogen content in seston and zooplankton were measured using a Flash EA 1112 NC Soil/MAS 200 elemental analyzer (ThermoQuest), as described in Gladyshev et al. (2007). The content of phosphorus in seston and zooplankton was estimated following the conventional photocolorimetric method (Murphy & Riley, 1962).

We analyzed the fatty acid contents of the seston and zooplankton using protocols from Gladyshev et al. (2015). Fatty acid methyl esters (FAMEs) were analyzed and identified using a gas chromatograph‐mass spectrometer (6890/5975C, “Agilent Technologies,” Santa Clara, CA, USA).

### 2.4 Primary and secondary production and the transfer efficiency of essential substances

The gross primary production (GPP) was estimated by the chlorophyll fluorescence method with DCMU (3‐(3,4‐dichlorophenyl)‐1,1‐dimethylurea), as described in Gaevsky et al. (2000). The vertical distribution of phytoplankton and PAR was measured in situ, and in the laboratory, we estimated the potential photochemical activity of photosystem II of algae with DCMU using the FluoroProbe with Workstation 25. The detailed procedure of GPP measurements was described elsewhere (Gaevsky et al., 2000; Gladyshev et al., 2011; Karpowicz, Zieliński, et al., 2020). The conversion factor for GPP from mg O_2_ to mg C was 0.32 (Alimov, 1989).

The secondary production (SP) of crustacean zooplankton was calculated using regression models from Stockwell and Johansson (1997):

$$\text{SP}=10^{\left(‐0.23\log(M)‐0.73\right)}1.12MN$$

where SP is the daily production of crustacean zooplankton (µg DW L^−1^ day^−1^), *M* is the mean individual dry weight (µg), and *N* is the abundance (individuals/L). The dry weight of zooplankton was converted into carbon units using a 1/2.3 quotient (Alimov, 1989).

We calculated the production of each substance by multiplying GPP or SP by the percentage content of each substance. Therefore, we determined how much substance was produced in phytoplankton or zooplankton daily per unit of volume. The trophic transfer efficiency (TTE) of each element (C, N, P) and ω‐3 PUFA from phytoplankton to zooplankton was measured as a ratio SP × substance content/GPP × substance content expressed in %. As such, TTE was presented as the ratio of production of a substance per day per liter in zooplankton to that of phytoplankton (Gladyshev et al., 2011). For the TTE of PUFA, we used the sum of all ω‐3 acids (18:3n‐3, 18:4n‐3, 20:4n‐3, 20:5n‐3, 22:5n‐3, and 22:6n‐3) following Gladyshev et al. (2011).

### 2.5 Statistical analysis

Statistical analyses were performed with XLSTAT Ecology (Addinsoft). We used one‐way ANOVA to determine whether the TTEs of essential substances differed between lakes in the different trophic groups (oligotrophic, mesotrophic, eutrophic, and dystrophic). Then, Fisher LSD (least significant difference) test was applied to find all pairwise differences between means. Basic descriptive statistics were calculated and presented as box plots where the limits of the boxes are the first and third quartiles, crosses represent the means, and the central horizontal bars are the medians. Points above or below are outliers, and the whiskers represented min and max. Canonical correspondence analysis (CCA) was performed to analyze the fatty acid composition of seston and zooplankton in different trophic conditions. CCA was performed according to Legendre and Legendre (1998), using STATISTICA software, version 9.0 (StatSoft, Inc.). FA levels (% of total fatty acids) were used in the CCA as axis of the multidimensional space. The CCA was also used to present the composition of zooplankton in different trophic conditions. The similarity of rotifers and crustacean communities was presented by agglomerative hierarchical cluster analysis (AHC) based on the Bray–Curtis similarity matrix (Figure S1).

## 3 RESULTS

### 3.1 Phytoplankton and zooplankton communities

**FIGURE 1:**
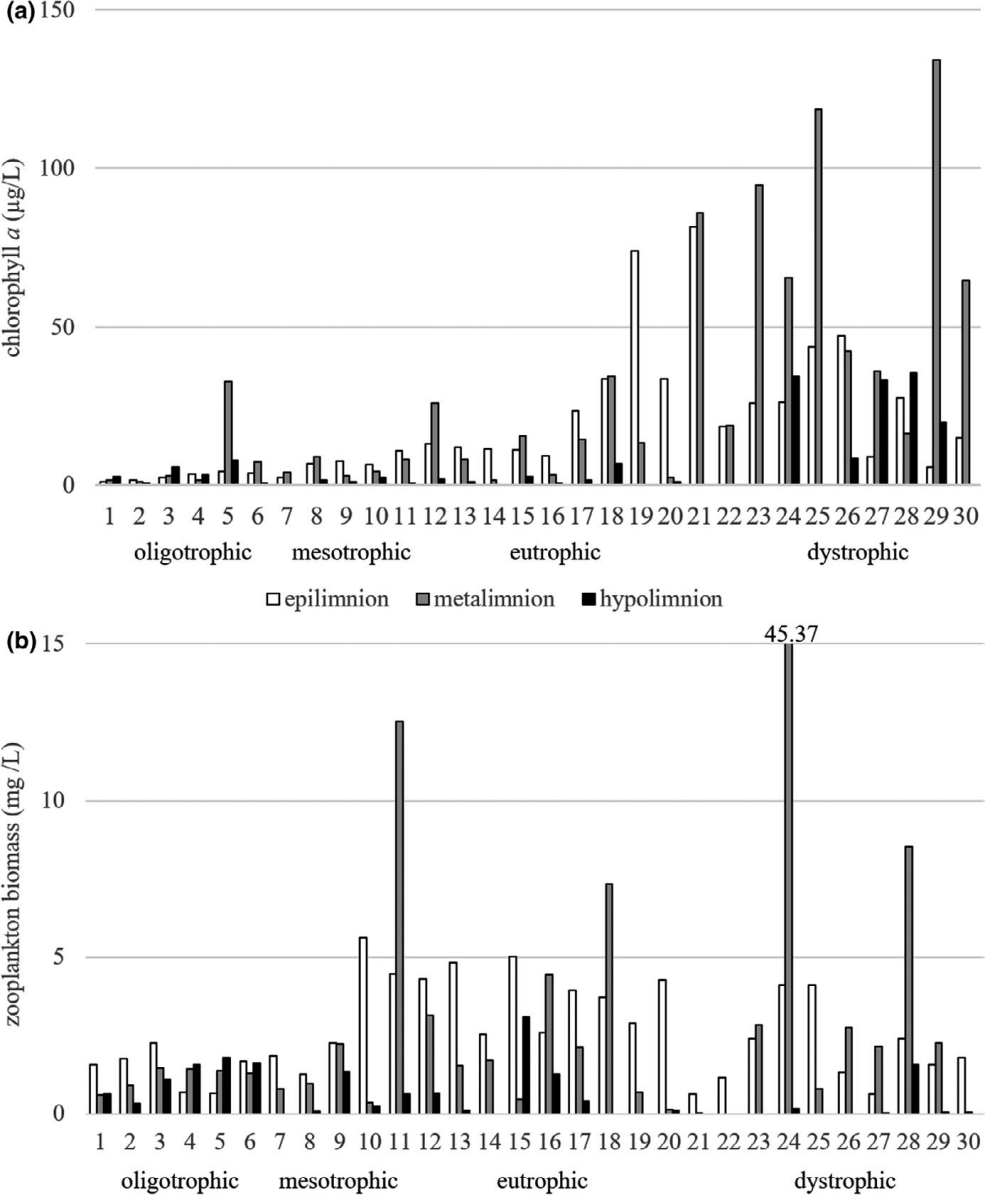
Chlorophyll *a* concentrations (a) and zooplankton biomasses (b) in the vertical profiles of the studied lakes

The chlorophyll *a* concentrations in the 30 lakes ranged from 0.21 to 134.0 µg/L. The lowest concentrations were in the oligotrophic and mesotrophic lakes (Figure 1a), with an average of 4.79 ± 7.95 µg/L and 5.97 ± 5.92 µg/L, respectively. These lakes most often had the highest concentrations of phytoplankton in the metalimnion or the hypolimnion (Figure 1a). The average chlorophyll *a* concentrations in eutrophic and dystrophic lakes was 14.62 ± 17.56 µg/L and 47.65 ± 35.76 µg/L, respectively. In eutrophic lakes, the maximum concentrations of phytoplankton were most often found in the epilimnion, while in dystrophic lakes they were most often found in the metalimnion (Figure 1a). The dominant phytoplankton taxa in most lakes were *Ceratium hirundinella* and *Dinobryon divergens*. The lakes with lower trophic status also had a greater relative abundances of algae from the genera *Cyclotella*, *Stephanodiscus*, and *Gloeotrichia*. Lakes of higher trophy had a greater relative abundances of *Planktothrix agardhii*, *Planktolyngbya limnetica*, *Limnotrix* sp., and *Microcystis* sp. The dystrophic lakes had greater relative abundances of *Gonyostomum* *semen*, *Cryptomonas* sp., and *Mougeotia* species.

Zooplankton biomass ranged from 0.01 (lake no. 27—hypolimnion) to 45.37 mg/L (Figure 1b) due to the mass development of *Asplanchna priodonta* (90% of the total zooplankton biomass). The lowest biomass of zooplankton was observed in oligotrophic lakes with an average of 1.21 ± 0.55 mg/L. Zooplankton biomass was relatively uniform throughout the vertical profile in oligotrophic lakes (Figure 1b). The average zooplankton biomass in mesotrophic and eutrophic lakes was 2.36 ± 2.82 mg/L and 2.53 ± 1.98 mg/L, respectively. However, the plankton distribution in the vertical profile was not uniform in these lakes (Figure 1b). The total biomass and vertical distribution of zooplankton varied in dystrophic lakes (Figure 1b). Shallow dystrophic lakes were characterized by low zooplankton biomass, while deeper dystrophic lakes (no. 24, 26, 27, 28, 29) had higher zooplankton biomass in the metalimnion (Figure 1b).

**FIGURE 2:**
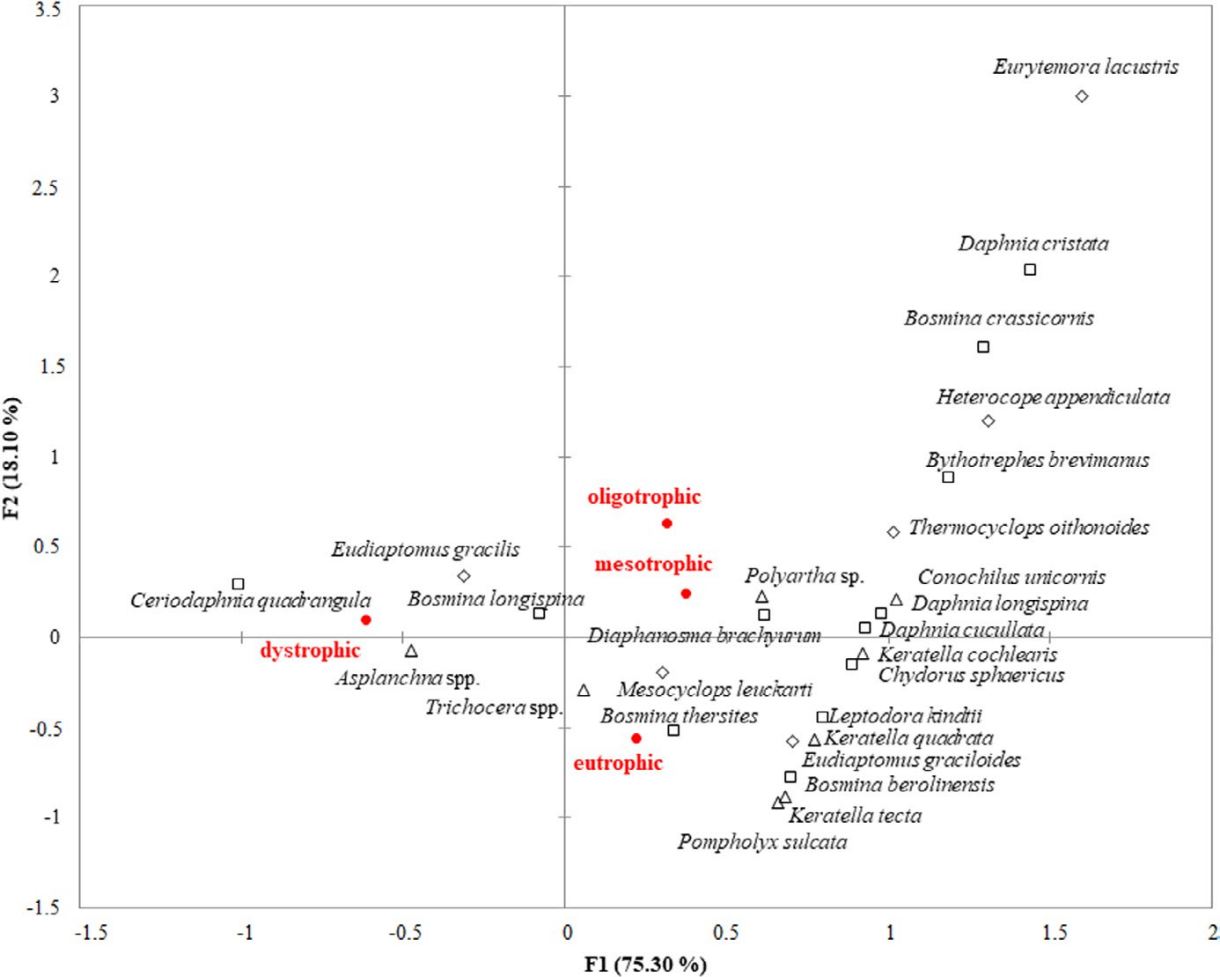
Dominant zooplankton species in different trophic conditions visualized by the CCA map. Cladocera marked with squares, Copepoda marked with rhombuses, and Rotifera marked with triangles

The crustacean communities had higher similarities than the rotifer communities in the studied lakes (Figure S1). Only the crustacean communities in dystrophic lakes were different from the other lakes (Figure S1B). The zooplankton communities in dystrophic lakes were dominated by *Asplanchna priodonta*, *Ceriodaphnia quadrangula,* and *Eudiaptomus gracilis* (Figure 2). Other species that frequently occurred in dystrophic lakes were *Bosmina longispina*, *Mesocyclops leuckarti*, *Diaphanosoma brachyurum*, and *Conochiloides dossuarius*. The zooplankton communities in other lakes (from oligotrophic to eutrophic) were generally dominated by *Daphnia* *cucullata*, *Diaphanosoma brachyurum*, *Thermocyclops oithonoides, Mesocyclops leuckarti*, and *Eudiaptomus graciloides*. However, there were differences in zooplankton structure related to trophic status. Lakes with higher trophic status had greater relative abundances of *Bosmina thersites*, *Bosmina berolinensis*, *Chydorus sphaericus*, *Pompholyx sulcatata*, *Keratella* spp., and *Trichocera* spp. (Figure 2), while lakes with lower trophic status had greater relative abundances of *Daphnia* *cristata*, *Daphnia* *longispina*, *Bosmina crassicornis*, *Eurytemora lacustris*, *Heterocope appendiculata*, *Bythotrephes brevimanus,* and *Conochilus unicornis* (Figure 2).

### 3.2 Elemental composition of phytoplankton and zooplankton

**FIGURE 3:**
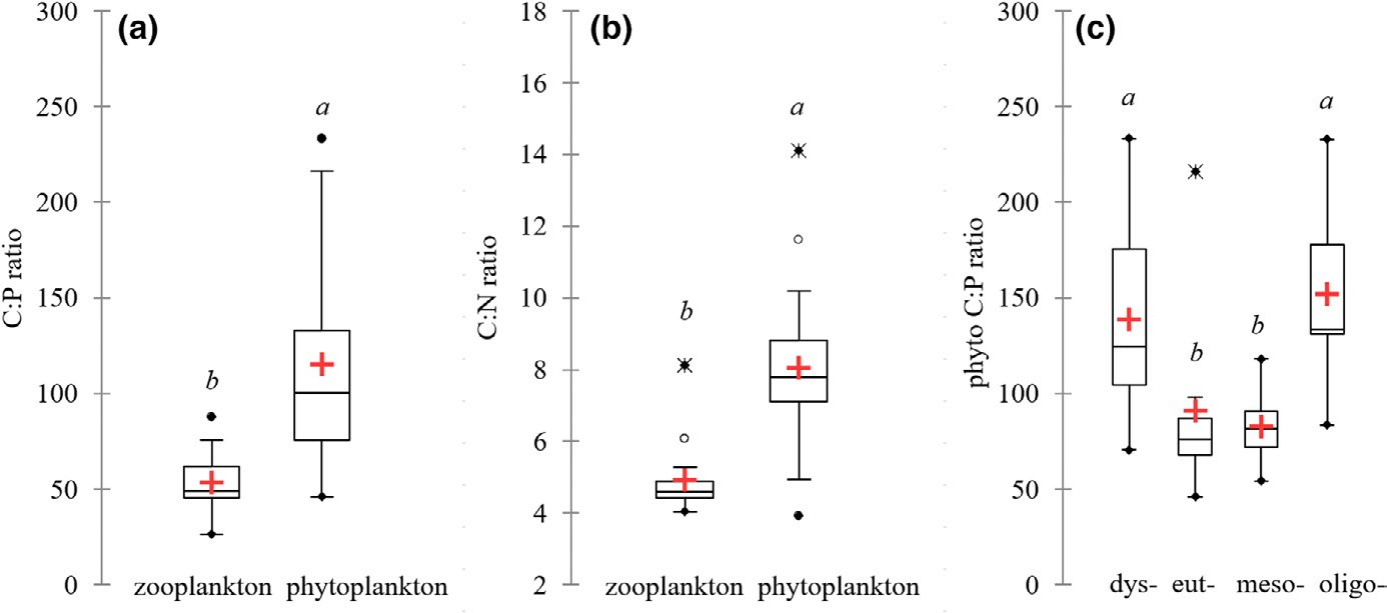
Zooplankton and phytoplankton nutrient content as a C:P ratio (a), C:N ratio (b), and differences in C:P ratio of phytoplankton in different trophic conditions (c). The different letters (*a*, *b*) above the box plots denote significantly different values at *p* < .05 and the same letters denote no statistically significant differences

**FIGURE 4:**
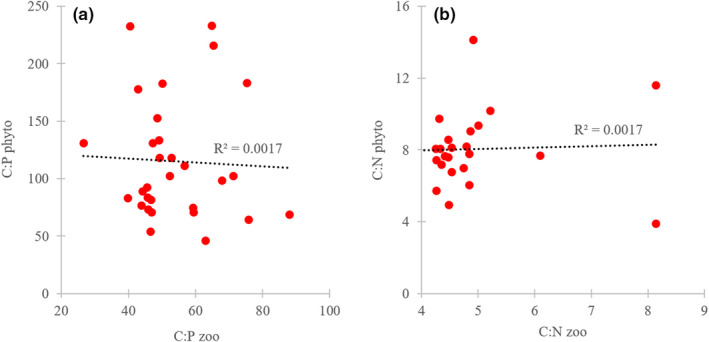
Relationship between elemental composition of phytoplankton and zooplankton, as a C:P ratio (a) and C:N ratio (b)

The elemental composition of zooplankton did not differ between the different trophic conditions, and the average contents of carbon, nitrogen, and phosphorus in zooplankton across all lake were 47.7 ± 1.7%, 9.9 ± 1.4%, and 0.9 ± 0.2. There were statistically significant differences between nitrogen and phosphorus content in phytoplankton and zooplankton (Figure 3). The C:P ratio of zooplankton was 53.7 ± 13.0, while the C:P ratio of phytoplankton was 115.2 ± 52.6 (Figure 3a). The large differences in C:P ratios of phytoplankton were related to the trophic status (Figure 3c). The highest C:P ratios of phytoplankton were observed in dystrophic and oligotrophic lakes, while significantly lower ratios occurred in eutrophic and mesotrophic lakes (Figure 3c). The mean C:N ratio of zooplankton was 4.9 ± 1.0, while in phytoplankton it was 8.0 ± 2.1 (Figure 3b). There were no differences in C:N ratios of phytoplankton and zooplankton based on the different trophic conditions. Thus, there was a large mismatch between the nitrogen and phosphorus content of phytoplankton (low) and zooplankton (high). Additionally, we did not observe a significant relationship between the phosphorus and nitrogen content in phytoplankton and zooplankton (Figure 4), which indicated that food quality did not affect the elemental composition of zooplankton.

### 3.3 Fatty acid composition of phytoplankton and zooplankton

**FIGURE 5:**
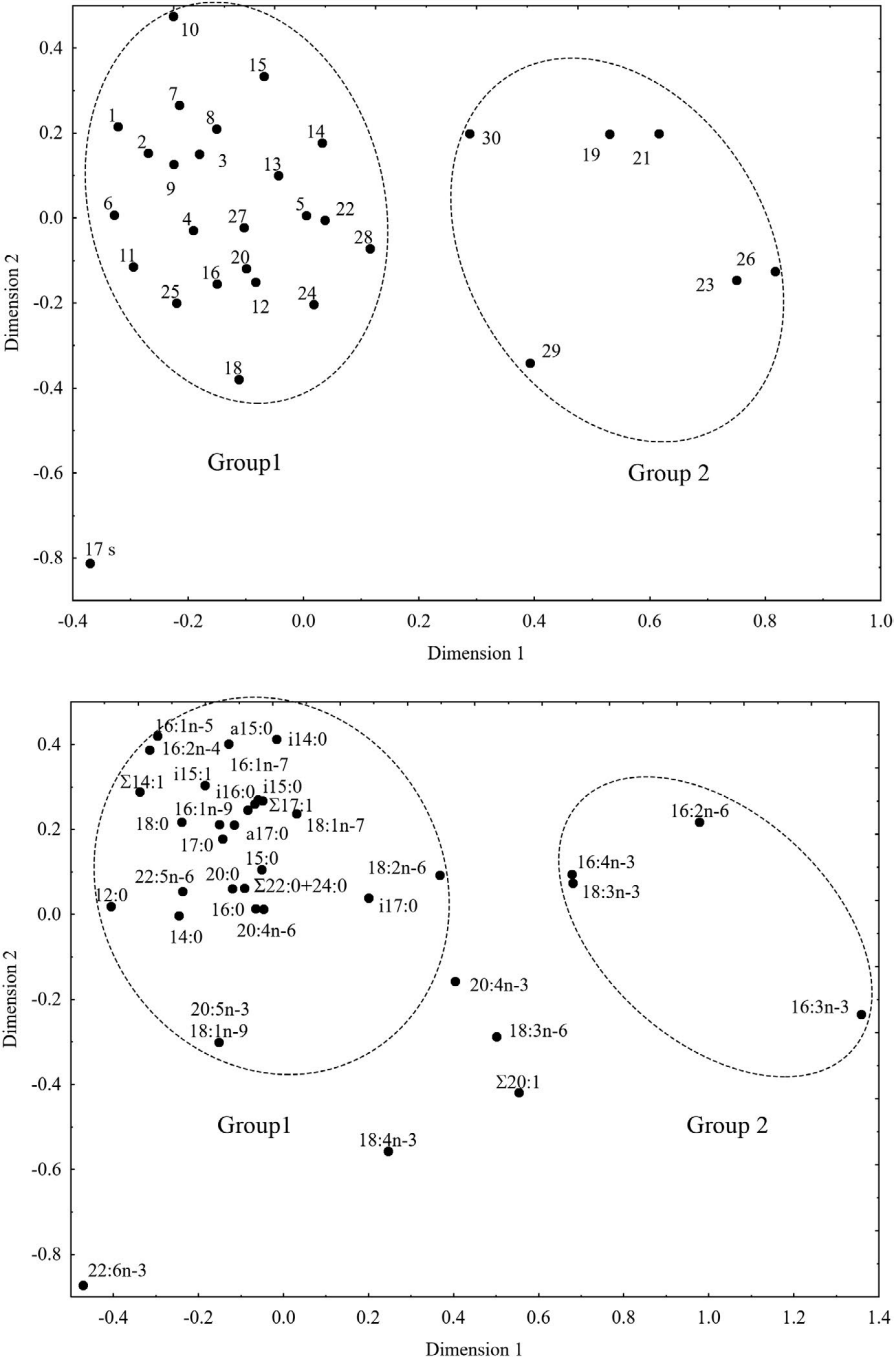
Canonical correspondence analysis of fatty acids composition (% of total FAs) of phytoplankton in studied lakes. The upper plot present studied lakes and the lower plot presents fatty acids markers

The canonical correspondence analysis (CCA) of the fatty acid composition of seston explained 29.3% of inertia along Dimension 1 and 17.5% along Dimension 2 (Figure 5). Group 1 was comprised of 23 lakes that were characterized by Group 1 fatty acids, which included markers of bacteria (i14:0, Σ14:1, i15:0, a15:0, i15:1, 15:0, i16:0, i17:0, a17:0, 17:0, Σ17:1, 18:1n‐7), detritus (16:0, 18:0, 20:0, Σ22:0 + 24:0), terrestrial (allochthonous) organic matter (18:2n‐6, 20:4n‐6), and diatom algae (16:1n‐7, 16:2n‐4, 20:5n‐3) (Figure 5). Group 2 was comprised of 6 lakes and was characterized by Group 2 fatty acids, which included markers of green algae and/or cyanobacteria (16:2n‐6, 16:3n‐3, 16:4n‐3, 18:3n‐3) (Figure 5). Only one lake (no. 17) was far from both groups and was separated by the marker of dynophyts, 22:6n‐3 (Figure 5). Indeed, this lake had the highest content of 22:6n‐3, 85.5 μg/L, as well as 20:5n‐3, 39.85 μg/L. In other lakes, average contents of 22:6n‐3 and 20:5n‐3 in seston were 5.56 ± 0.73 μg/L and 3.62 ± 1.04 μg/L, respectively. The marker of cryptophytes and other flagellates, 18:4n‐3, had an intermediate position in the two dimensions of the CCA (Figure 5).

**FIGURE 6:**
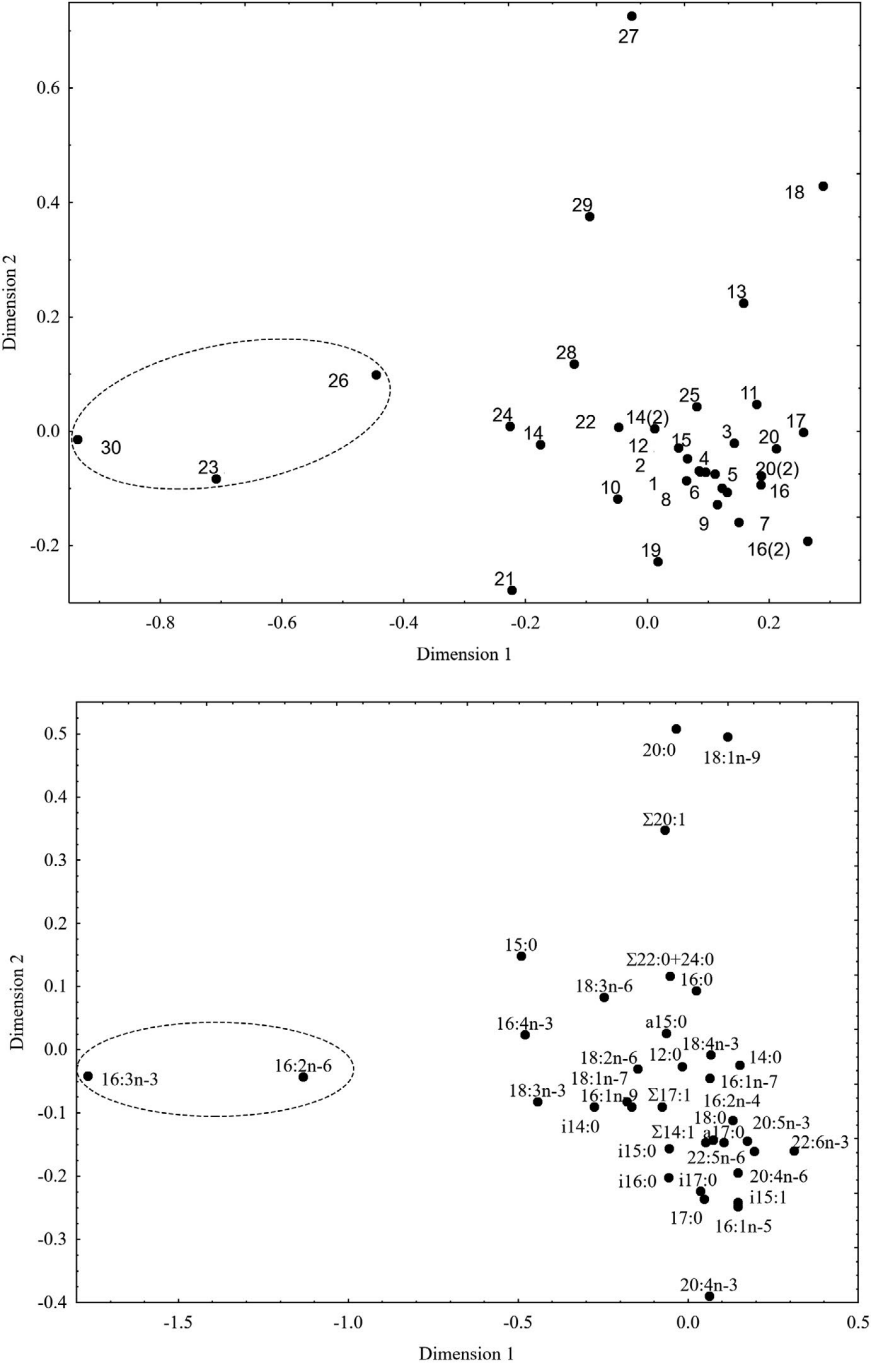
Canonical correspondence analysis of fatty acids composition (% of total FAs) of zooplankton in studied lakes. The upper plot present studied lakes and the lower plot presents fatty acids markers

The CCA of zooplankton fatty acid composition explained 35.3% of inertia along Dimension 1 and 19.1% along Dimension 2 (Figure 6). In contrast to seston, only three lakes separated from the other lakes (Figure 6). This group partly coincided with Group 2 in seston (Figure 5) and was separated by a part of the same markers of green algae (16:2n‐6, 16:3n‐3) (Figures 5 and 6). Except for this small group, there was no explicit separation of zooplankton FA markers in the studied lakes.

The FA composition in seston and zooplankton showed correspondence in only three of the dystrophic lakes (no. 23, 26, 30). Indeed, characteristics of seston in these three lakes were comparatively showed high levels of the markers of green algae, 16:2n‐6 and 16:3n‐3. Correspondently, there were high levels of these biomarkers in the zooplankton in these lakes. In the other lakes, there was no such correspondence between the FA composition of seston and zooplankton.

### 3.4 Primary and secondary production

**FIGURE 7:**
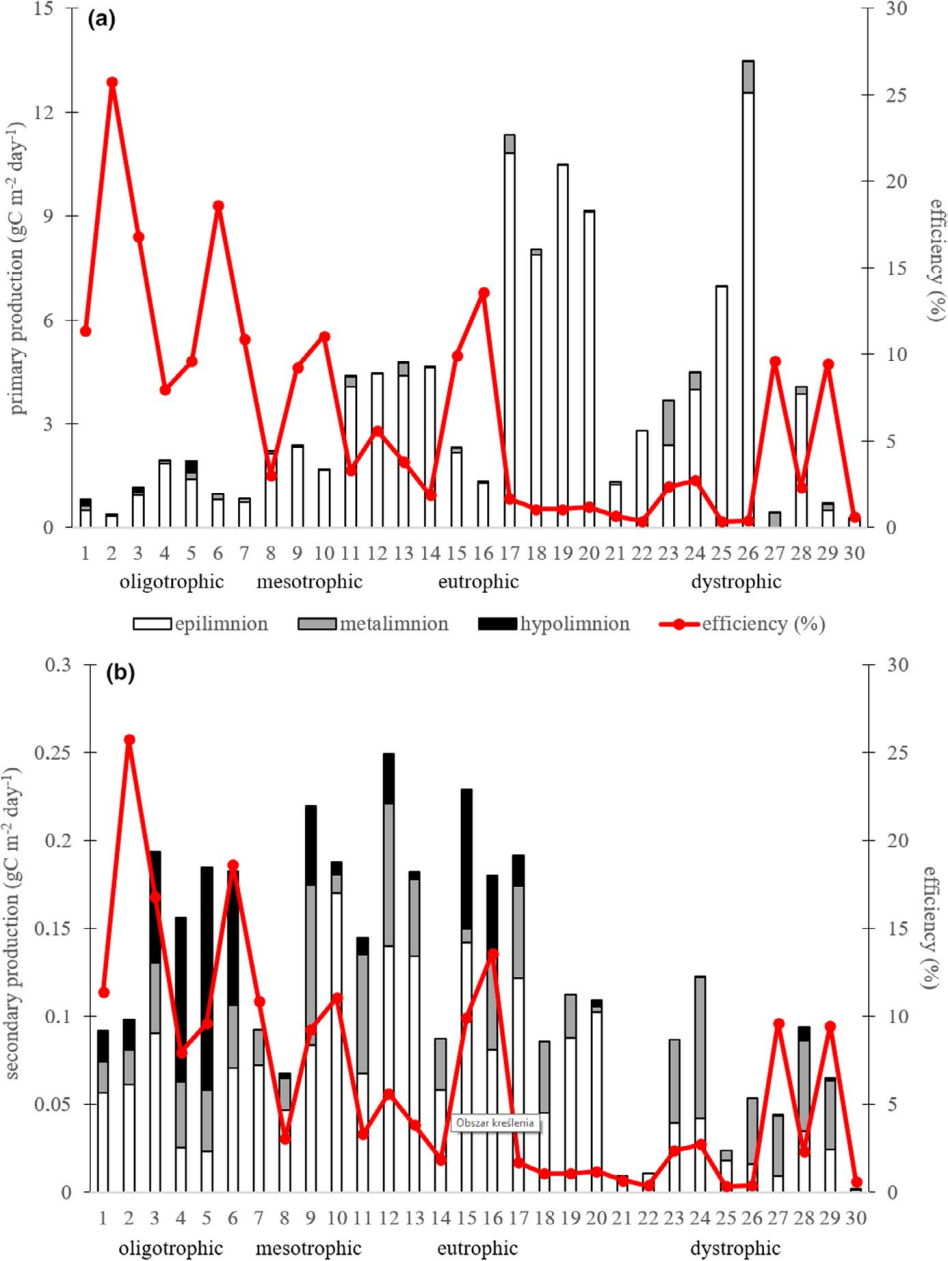
Primary production (a) and secondary production (b) in the whole water profile of lakes with different trophic status, with the efficiency of carbon transfer as a red line

The gross primary production (GPP) ranged from 0.29 to 13.47 gC m^−2^ day^−1^ (Figure 7a), and there were some differences related to the trophic status (*F*_3.26_ = 3.5; *p* = .029). The highest GPP was in eutrophic lakes (6.51 ± 3.53 gC m^−2^ day^−1^) and in dystrophic lakes (3.82 ± 3.80 gC m^−2^ day^−1^). However, there were large differences in the GPP between the dystrophic lakes, and some of the lowest and highest values of GPP were found in these lakes (Figure 7a). The lowest GPP was in oligotrophic (1.25 ± 0.62 gC m^−2^ day^−1^) and in mesotrophic lakes (2.42 ± 1.36 gC m^−2^ day^−1^) (Figure 7a). The epilimnetic primary production was much higher than metalimnetic and hypolimnetic, and it accounted for more than 90% of the total GPP (Figure 7a). The one exception to this was oligotrophic lakes where the metalimnetic and hypolimnetic algae production were an important part of total GPP (Figure 7a).

The secondary production (SP) ranged from 0.002 to 0.25 gC m^−2^ day^−1^ (Figure 7b). The secondary production was generally more homogenous in the vertical profile, with similar SP in the epilimnion and lower water layers (Figure 7b). The lowest SP was in the dystrophic lakes (0.05 ± 0.4 gC m^−2^ day^−1^), where most of the SP was related to the metalimnetic zooplankton production (Figure 7b). There were no significant differences between SP in oligotrophic, mesotrophic and eutrophic lakes, where average SP was 0.15 ± 0.5 gC m^−2^ day^−1^ (Figure 7b).

### 3.5 The efficiency of transfer of essential substances from phytoplankton to zooplankton

**FIGURE 8:**
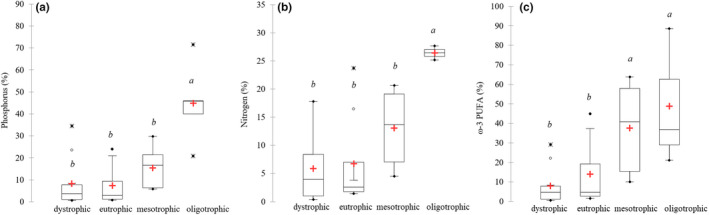
Transfer efficiency (%) of phosphorus (a), nitrogen (B), and sum of ω‐3 PUFA (c) from phytoplankton to zooplankton in lakes with different trophic status. The different letters (*a*, *b*, *c*) above the box plots denote significantly different values at *p* < .05 and the same letters denote no statistically significant differences

The TTE of carbon from phytoplankton to zooplankton ranged from 0.34% to 25.8% (Figure 7) with an average of 6.55 ± 6.40%. The trophic conditions significantly influenced the TTE of carbon (*F*_3.26_ = 6.65; *p* = .002), which decreased with increasing trophic status (Figure 7). The highest average TTE of carbon was in oligotrophic lakes (14.31 ± 6.50%), and the lowest in dystrophic and eutrophic lakes where the TTE of carbon was 2.89 ± 3.45% and 4.28 ± 4.49%, respectively (Figure 7). However, among dystrophic and eutrophic lakes there was also efficient carbon transfer in lakes no. 15, 16, 27, 29 (Figure 7). The TTE of phosphorus ranged from 0.57% to 71.57% with an average of 15.82 ± 17.61%. There were large differences in phosphorus transfer in relation to trophic conditions (*F*_3.26_ = 12.61; *p* < .0001), with the highest transfer in oligotrophic lakes (Figure 8a). The average TTE of nitrogen was 9.82 ± 9.12%, and there were differences related to different trophic conditions (*F*_3.26_ = 4.92; *p* = .011). The highest nitrogen efficiency was in oligotrophic lakes, while the lowest was in eutrophic and dystrophic lakes (Figure 8b). The average TTE of ω‐3 PUFA was 20.90 ± 23.60% (excluding 3 evident artefacts with TTE >100%, which likely appeared because the contents of ω‐3 PUFA in seston were too low for reliable measurements), and there were differences related to different trophic conditions (*F*_3.26_ = 5.55; *p* = .005). The highest ω‐3 PUFA efficiencies were in oligotrophic and mesotrophic lakes, while the lowest were in eutrophic and dystrophic lakes (Figure 8c).

In conclusion, there were differences in transfer efficiencies between essential substances. The average TTE of C, N, P, and ω‐3 PUFA was 6.55%, 9.82%, 15.82%, and 20.90%, respectively. There were also large differences in transfer efficiencies between trophic conditions, with the highest efficiencies in oligotrophic lakes and lowest in dystrophic and eutrophic lakes. Comparing the extreme TTE values for C, N, P, and ω‐3 PUFA we found that the difference could be as much as 76‐fold, 73‐fold, 125‐fold, and 150‐fold between lakes, respectively.

## 4 DISCUSSION

Plankton transfer energy and matter from primary producers to higher trophic levels and thus play a pivotal role in the biogeochemical cycling in lakes. We found that different essential substances were transferred from phytoplankton to zooplankton with varying efficiencies across a trophic gradient. Most of the previous research on TTE is related to bulk carbon because phytoplankton converts carbon from inorganic to organic, which is incorporated into zooplankton biomass (Pauly & Christensen, 1995; Schulz et al., 2004). The results of our study showed that the average TTE of bulk carbon between phytoplankton and zooplankton in the middle of summer was 6.55 ± 6.40%, which is in accordance with a general paradigm that about 10% of organic carbon production of one trophic level is incorporated into new biomass on the next trophic level (Lindeman, 1942). Also, data on annual primary and secondary production suggest that TTE varies around 5%–10% (Gladyshev et al., 2011; Lacroix et al., 1999; Schulz et al., 2004), but in the middle of summer can be much lower in highly eutrophic lakes (Gladyshev et al., 2011), and much higher in oligotrophic lakes (Schulz et al., 2004). The direct measurements of carbon transfer efficiency from bacteria and algae to zooplankton in a subtropical eutrophic lake in Florida showed much lower TTE, which ranged between 0.1% to 1% and indicated a large loss of carbon from the food web dominated by copepod and cyanobacteria (Havens et al., 2000). Thus, the TTE of carbon can be highly variable in freshwater lakes, and there is evidence that an efficient system can support 25 times more biomass of zooplankton than highly eutrophic lakes (Brett & Müller‐Navarra, 1997).

We found that the TTE of carbon in our 30 study lakes ranged from 0.34% to 25.8%, which represented a 76‐fold difference across the trophic gradient. The highest TTE of carbon was observed in oligotrophic lakes, while the lowest was observed in dystrophic and eutrophic lakes. The oligotrophic lakes were dominated by larger zooplankton, while zooplankton in highly eutrophic and dystrophic lakes were dominated by small species. The well‐oxygenated hypolimnion in oligotrophic lakes creates favorable habitat for large cold‐water species and increases zooplankton species richness, which further promotes the effective transfer of matter (Karpowicz, Ejsmont‐Karabin, et al., 2020). Decreases in TTE can also be attributed to an increase in inedible algae in the eutrophic lakes, which were dominated by filamentous cyanobacteria, while in dystrophic lakes there was commonly mass development of the large raphidophyte *Gonyostomum* *semen* (Pęczuła et al., 2018). Therefore, our study indicated that different disturbances like eutrophication and dystrophication similarly decrease the TTE of matter between phytoplankton and zooplankton.

We found a similar pattern for other essential substances (N, P ω‐3 PUFA) where TTE decreased with increasing trophic status. Furthermore, there were differences between the TTE of different substances, where phosphorus and ω‐3 PUFA had about two times higher TTE than carbon and nitrogen. This is in accordance with Gladyshev et al. (2011) who stated that essential PUFAs were transferred from phytoplankton to zooplankton with about twice the efficiency of bulk carbon. The high TTE of phosphorus in our study could be related to the elemental ratio mismatch between phytoplankton (low P) and zooplankton (high P). To maintain stoichiometric balance zooplankton can accumulate substances that are in shortage and excrete substances that are in excess (Hessen, 1992; Schoo et al., 2013; Sterner, 1997; Sterner et al., 1998). Laboratory experiments showed that the excretion of P by *Daphnia* became zero when C:P ratio in the food was above 300 (Olsen et al., 1986). The results of our study revealed that the TTE of phosphorus from phytoplankton to zooplankton was about two times higher than carbon, which indicated that zooplankton effectively accumulated phosphorus. The nitrogen was transferred with similar efficiencies as carbon in our study. Generally, the N limitation of freshwater zooplankton is not significant because phytoplankton C:N ratios are less variable than C:P ratios (Malzahn et al., 2010).

The elemental difference between the primary producers and primary consumers affected the biochemical cycles. Phytoplankton and zooplankton require common elements like C, O, and H to build up their biomass, but also nutrients such as N and P (Sterner & Elser, 2002). Phytoplankton is an important link in the transformation of nutrients from inorganic (i.e., NO_3_^−^, NH_4_^+^, PO_4_^3−^) to organic forms, thus reducing the amount of reactive nutrients in the water (Karpowicz, Zieliński, et al., 2020). Zooplankton then transfers this matter to higher trophic levels, which plays an important role in nutrient cycles. The food resources for zooplankton are not only phytoplankton but also bacteria and other particles collectively labeled “seston,” and as a result, C:P ratio varies greatly between 100 and 1,000. High C:P ratios of phytoplankton are frequently observed in summer (Malzahn et al., 2010), but in our study seston C:P ratios were relatively low (115 ± 53), which indicated relatively high food quality for zooplankton. Furthermore, the C:P ratio of seston in our study was related to the trophic status. The lowest phosphorus content in seston was found in oligotrophic and dystrophic lakes, while the highest phosphorus content in seston was found in mesotrophic and eutrophic lakes. In zooplankton, there were no differences in the C:P and C:N ratios between different trophic conditions. This indicates a stable elemental composition of zooplankton, despite the differences in community structure and dominant taxa between the lake groups. The content of C, N, and P in zooplankton was 47.7 ± 1.7%, 9.9 ± 1.4%, and 0.9 ± 0.2, respectively. These represent typical elemental compositions of freshwater zooplankton found in temperate lakes (Hessen, 2008; Hessen et al., 2013; Karpowicz, Feniova, et al., 2019) and in the Baltic Sea (Walve & Larsson, 1999). We have additionally highlighted the role of zooplankton in the accumulation of phosphorus and ω‐3 PUFA. The finding that phosphorus can be transferred with higher efficiency than carbon and nitrogen and thereby accumulate in zooplankton is very important for biogeochemical cycles and the understanding of some ecological processes.

Other biomolecules that are essential to the zooplankton are polyunsaturated fatty acids (PUFA) of the ω‐3 family with 18–22 carbon atoms. Zooplankton, in contrast to algae, cannot synthesize de novo the parent acid of this family, 18:3n‐3, and efficiently convert it to the longer chain PUFA (Bell & Tocher, 2009; Lands, 2009). The results of our study revealed that the essential ω‐3 PUFA were transferred from phytoplankton to zooplankton with about ten times higher efficiency than bulk carbon. This indicated that the essential ω‐3 PUFA could be assimilated by zooplankton with maximum efficiency during the peak of the summer stagnation. The results of Gladyshev et al. (2011) indicated that the average TTE of PUFA over the whole growing season was about twice as high as that of bulk carbon. The results from seasonal changes in the accumulation of PUFA in zooplankton indicated a significant increase in the accumulation of the docosahexaenoic acid (22:6n‐3, DHA) during late summer and autumn. Authors have previously linked it with the dominance of copepods which increasingly accumulated DHA for overwintering (Hartwich et al., 2013). The other reason for the high efficiency of ω‐3 PUFA in our study could be the effect of the seasonal succession of plankton. The spring phytoplankton is often dominated by diatoms that are rich in eicosapentaenoic acid (20:5n‐3, EPA), whereas diatoms are much less abundant in the summer (Hartwich et al., 2012). Therefore, the accumulation of EPA by zooplankton should increase. Also, abiotic factors such as temperature or light intensity are negatively correlated with PUFA concentrations in seston (Gladyshev et al., 2010; Piepho et al., 2012; Thompson et al., 1990, 1992). We found similar differences in TTE of essential PUFA and nutrients in different trophic conditions, with the highest efficiency in oligotrophic and the lowest in dystrophic and eutrophic lakes. This indicates that disturbances such as eutrophication and dystrophication similarly reduced the transfer efficiency of essential substances in the planktonic food web. The results of our previous study indicated that the presence of oxygen in the hypolimnion is an important factor that promotes the efficiency of matter transfer (Karpowicz, Ejsmont‐Karabin, et al., 2020).

The results of this study showed that there was not a clear relationship between the fatty acid composition of seston and zooplankton. This likely means that zooplankton generally had a high degree of feeding selectivity, and consumed food items that included microalgae, and other components of seston with an appropriate nutritive quality only. Deep lakes are characterized by large differences in the vertical distribution of phytoplankton (Camacho, 2006; Miracle et al., 1993) and crustacean zooplankton (Karpowicz, Ejsmont‐Karabin, et al., 2019; Rosenzweig, 1991). The other reason why the fatty acid composition of seston was not transferred to zooplankton biomass in our study could be the fact the transfer is not instant but has a time lag that may not have been captured by our snapshot sampling approach (Gladyshev et al., 2011; Taipale et al., 2009).

## 5 CONCLUSIONS

The results of our study revealed that different substances were transferred from phytoplankton to zooplankton with varying efficiencies. The average TTE of C, N, P, and ω‐3 PUFA was 6.55%, 9.82%, 15.82%, and 20.90%, respectively. There were also large differences in transfer efficiencies between trophic conditions, with the highest transfer efficiencies in oligotrophic lakes and the lowest in dystrophic and eutrophic lakes. This indicated that different disturbances like eutrophication and dystrophication similarly decrease the TTE of essential substances between phytoplankton and zooplankton. The large mismatch between elemental and biochemical compositions of zooplankton and their food may further promote the accumulation of these substances that are in shortage.

## CONFLICT OF INTEREST

The authors declare no conflict of interest.

## AUTHOR CONTRIBUTIONS

**Maciej Karpowicz:** Conceptualization (equal); Formal analysis (equal); Investigation (lead); Project administration (lead); Visualization (lead); Writing‐original draft (lead). **Irina Feniova:** Conceptualization (supporting); Funding acquisition (lead); Writing‐review & editing (supporting). **Michail I. Gladyshev:** Formal analysis (supporting); Validation (lead); Writing‐review & editing (supporting). **Jolanta Ejsmont‐Karabin:** Investigation (supporting). **Andrzej Górniak:** Investigation (supporting). **Nadezhda N. Sushchik:** Investigation (supporting). **Olesya V. Anishchenko:** Validation (supporting). **Andrew R. Dzialowski:** Supervision (lead); Writing‐review & editing (lead).

## Supporting information

Supplementary Material

## Data Availability

Data from this manuscript are available: Dryad https://doi.org/10.5061/dryad.d51c5b034.
