# Supplementary material for: Transfer efficiency of carbon, nutrients, and polyunsaturated fatty acids in planktonic food webs under different environmental conditions
Source: Ecol Evol. 2021 May 18;11(12):8201–14. doi: 10.1002/ece3.7651 (PMC8216980; doi:10.1002/ece3.7651)
Supplement: Supplementary file 1 — Supplementary Material [file ECE3-11-8201-s001.docx]

**Supplementary Materials**

**Table S1.** Morphometric and trophic characteristics of the studied lakes in NE Poland. SDV - Secchi disc visibility; TSI – Carlson trophic state index; HDI - hydrochemical dystrophy index; nd. – no data.

| **no.** | **lakes** | **latitude (N)** | **longitude (E)** | **surface (ha)** | **max depth (m)** | **avg. depth (m)** | **SDV (m)** | **TSI** | **HDI** | **trophic conditions** |
| --- | --- | --- | --- | --- | --- | --- | --- | --- | --- | --- |
| 1 | Białe Filipowskie | 54.194757 | 22.653199 | 132.4 | 52.0 | 17.1 | 7.5 | 29.6 | 35.1 | oligotrophic |
| 2 | Gaładuś | 54.169779 | 23.421463 | 728.6 | 54.8 | 12.7 | 5.7 | 33.8 | 35.3 | oligotrophic |
| 3 | Serwy | 53.914224 | 23.214309 | 460.3 | 41.5 | 14.6 | 3.8 | 38.5 | 35 | oligotrophic |
| 4 | Leleskie | 53.640855 | 20.845268 | 423.5 | 49.5 | 12.2 | 5.5 | 39.7 | 37.3 | oligotrophic |
| 5 | Jegocin | 53.664689 | 21.697784 | 127.4 | 36.1 | 9.0 | 7.0 | 39.7 | 37.7 | oligotrophic |
| 6 | Buwełno | 53.887375 | 21.865920 | 360.3 | 49.1 | 12.4 | 2.4 | 42.4 | 30.7 | mesotrophic |
| 7 | Majcz Wielki | 53.778685 | 21.455861 | 163.5 | 16.4 | 6.0 | 2.7 | 42.8 | 34.1 | mesotrophic |
| 8 | Kuc | 53.820273 | 21.407373 | 98.8 | 28.0 | 8.0 | 4.0 | 43.5 | 34.7 | mesotrophic |
| 9 | Probarskie | 53.824763 | 21.373934 | 201.4 | 31.0 | 9.2 | 3.8 | 46.3 | 33.6 | mesotrophic |
| 10 | Brzozolasek | 53.615561 | 21.737652 | 155.9 | 17.2 | 5.1 | 1.2 | 49.4 | 36.0 | mesotrophic |
| 11 | Mikołajskie | 53.783641 | 21.589658 | 497.9 | 25.9 | 11.2 | 2.0 | 49.9 | 34.8 | mesotrophic |
| 12 | Kalwa | 53.657656 | 20.788987 | 562.2 | 31.7 | 7.0 | 2.0 | 49.6 | 34.8 | mesotrophic |
| 13 | Jagodne | 53.922153 | 21.712378 | 942.7 | 37.4 | 8.7 | 2.2 | 50.3 | 29.6 | eutrophic |
| 14 | Boczne | 53.958124 | 21.745201 | 183.3 | 17.0 | 8.7 | 1.4 | 51.1 | 32.2 | eutrophic |
| 15 | Wiartel | 53.599383 | 21.693855 | 178.6 | 29.0 | 4.5 | 1.6 | 51.7 | 37.7 | eutrophic |
| 16 | Ryńskie | 53.911576 | 21.501917 | 670.8 | 50.8 | 10.0 | 1.0 | 52.5 | 34.3 | eutrophic |
| 17 | Kierźlińskie | 53.800811 | 20.743641 | 92.8 | 44.5 | 11.7 | 1.5 | 53.1 | 32.7 | eutrophic |
| 18 | Nidzkie | 53.575114 | 21.541653 | 1818.0 | 23.7 | 6.2 | 0.9 | 55.4 | 35.4 | eutrophic |
| 19 | Miłkowskie | 53.941745 | 21.871101 | 23.7 | 15.0 | 4.2 | 0.5 | 59.4 | 32.3 | eutrophic |
| 20 | Juno | 53.905898 | 21.294951 | 380.7 | 33.0 | 11.9 | 0.7 | 62.9 | 32.7 | eutrophic |
| 21 | Klimunt | 53.706380 | 21.448366 | 12.8 | 4.0 | 2.6 | 0.3 | 63.8 | 60.4 | dystrophic |
| 22 | Wesołek | 53.592903 | 21.511525 | 7.0 | 3.0 | nd. | 1.2 | 50.7 | 66.9 | dystrophic |
| 23 | Zdruźno | 53.636779 | 21.350683 | 6.8 | 5.0 | nd. | 1.8 | 51.2 | 69.8 | dystrophic |
| 24 | Gryżewskie | 53.723882 | 21.551170 | 4.3 | 5.0 | nd. | 1.8 | 50.7 | 71.4 | dystrophic |
| 25 | Sęczek | 53.728078 | 21.546483 | 3.8 | 3.5 | nd. | 0.9 | 56 | 72.2 | dystrophic |
| 26 | Bolkowskie | 53.721397 | 21.549049 | 2.9 | 5.0 | nd. | 1.2 | 53.7 | 73.2 | dystrophic |
| 27 | Kruczy Staw | 53.657748 | 21.417198 | 2.1 | 8.0 | nd. | 2.0 | 47.7 | 73.3 | dystrophic |
| 28 | Kruczek Duży | 53.660037 | 21.402607 | 4.2 | 4.0 | nd. | 1.5 | 51.3 | 74.2 | dystrophic |
| 29 | Kruczek Mały | 53.661550 | 21.406126 | 2.6 | 9.0 | nd. | 1.7 | 46.7 | 74.6 | dystrophic |
| 30 | Konopniak | 54.035222 | 23.183138 | 9.5 | 4.0 | nd. | 1.2 | 50.4 | 79.9 | dystrophic |


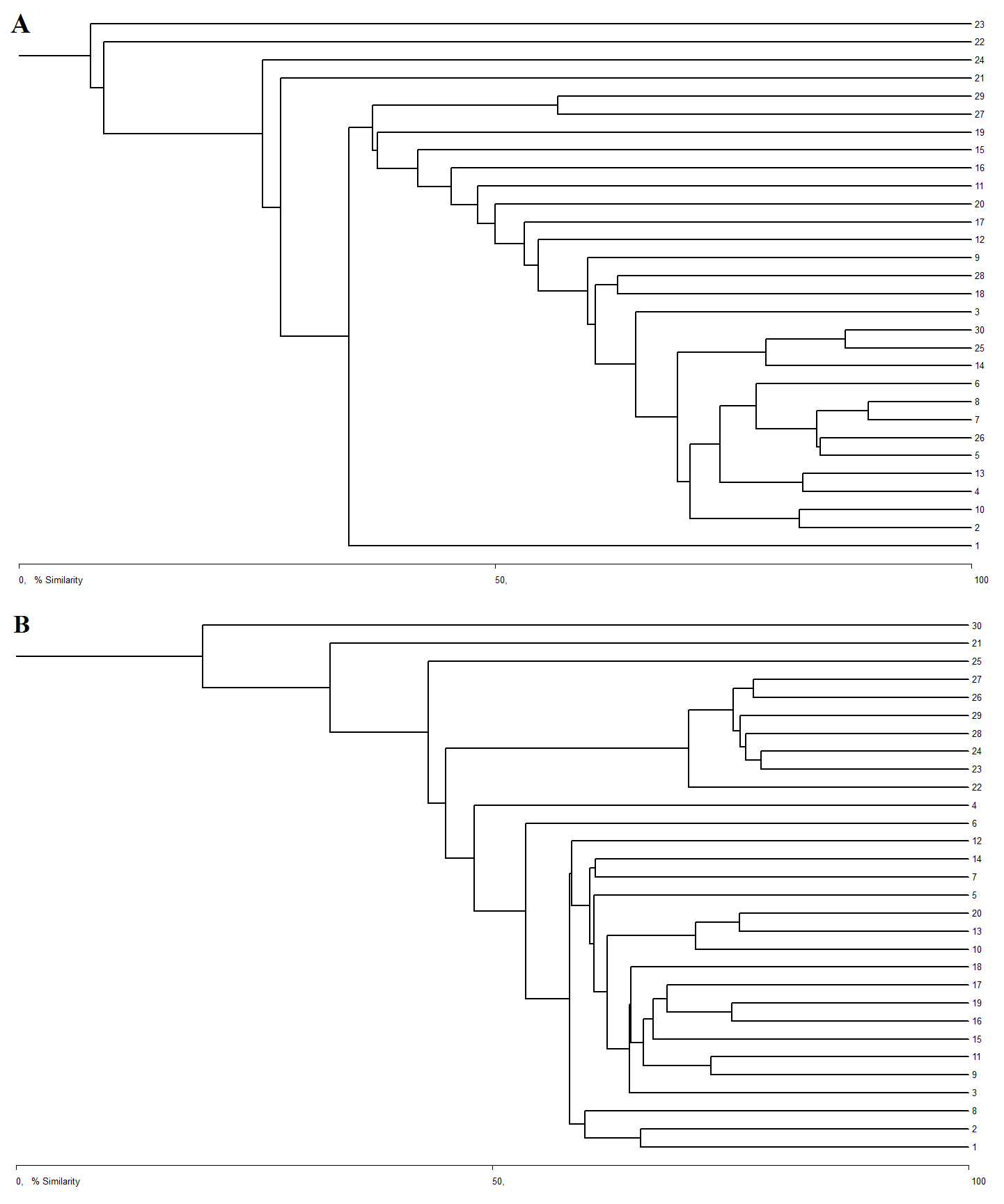


**Fig. S1.** The Bray-Curtis similarity matrix of rotifers (A) and crustacean (B) communities in the analyzed lakes based on the agglomerative hierarchical cluster analysis (AHC).
